# Quantitative shadow compensated optical coherence tomography of choroidal vasculature

**DOI:** 10.1038/s41598-018-24577-8

**Published:** 2018-04-24

**Authors:** Kiran Kumar Vupparaboina, Kunal K. Dansingani, Abhilash Goud, Mohammed Abdul Rasheed, Fayez Jawed, Soumya Jana, Ashutosh Richhariya, K. Bailey Freund, Jay Chhablani

**Affiliations:** 1 Surjana Center for Innovation, LV Prasad Eye Institute Hyderabad, Hyderabad, Telangana India; 2 Department of Electrical Engineering, Indian Institute of Technology Hyderabad, Hyderabad, Telangana India; 3 Department of Ophthalmology, University of Pittsburgh Medical Center, Pittsburgh, Pennsylvania USA; 4 Clinical Research, LV Prasad Eye Institute Hyderabad, Hyderabad, Telangana India; 5 Vitreo-retinal Service, LV Prasad Eye Institute Hyderabad, Hyderabad, Telangana India; 6 Truhlsen Eye Institute, University of Nebraska Medical Center, Omaha, NE United States; 7 Vitreous Retina Macula Consultants of New York, New York, New York, USA; 8 LuEsther T. Mertz Retinal Research Center, Manhattan Eye, Ear and Throat Hospital, New York, New York, USA

## Abstract

Conventionally rendered optical coherence tomography (OCT) images of the posterior segment contain shadows which influence the visualization of deep structures such as the choroid. The purpose of this study was to determine whether OCT shadow compensation (SC) alters the appearance of the choroid and the apparent choroidal vascularity index (CVI), an OCT-derived estimated ratio of luminal to total choroidal volume. All scans were shadow compensated using a previously published algorithm, binarized using a novel validated algorithm and extracted binarized choroid to estimate CVI. On 27 raw swept-source OCT volume-scans of healthy subjects, the effect of SC on CVI was established both qualitatively and quantitatively. In shadow compensated scans, the choroid was visualized with greater brightness than the neurosensory retina and the masking of deep tissues by retinal blood vessels was greatly reduced. Among study subjects, significant mean difference in CVI of −0.13 was observed between raw and shadow compensated scans. Conventionally acquired OCT underestimates both choroidal reflectivity and calculated CVI. Quantitative analysis based on subjective grading demonstrated that SC increased the contrast between stromal and luminal regions and are in agreement with true tissue regions. This study is warranted to determine the effects of SC on CVI in diseased eyes.

## Introduction

Optical coherence tomography acquires depth-resolved tissue reflectivity data using a scanning near-infrared light source. Data are represented as pixel or voxel arrays in which brightness encodes focal tissue reflectivity. Meaningful observation of the ocular posterior segment is possible because the tissues are sufficiently transparent to allow subsurface photon penetration yet sufficiently opaque and optically heterogeneous to return structurally relevant and informative reflections.

Imaging the choroid with OCT is challenging because of the masking effect of the relatively opaque retinal pigment epithelium (RPE). Enhanced depth imaging and averaging improve visualisation of the choroid on cross-sectional OCT scans but averaging requires multiple scan passes and prolongs acquisition times^1,2^. Swept-source OCT employs a tunable laser and allows fast acquisition of dense raster scans^3^. It also operates at longer wavelengths and achieves deeper tissue penetration so that the full thickness of the choroid is better visualized, albeit with persistent shadows from anterior structures^4^. Qualitative analysis of images produced using these strategies has yielded important information about diseases in which alterations in choroidal vascular architecture are considered etiological and has fed a growing interest in quantifying these changes using automated algorithms.

To evaluate the choroid quantitatively, various parameters including choroidal thickness, choroidal volume and more recently choroidal vascularity index (CVI), have been devised. Choroidal vascularity index is the ratio of luminal to total choroidal volume. Automated estimation of CVI has been reported by our group in healthy as well as diseased conditions such as high myopia, central serous chorioretinopathy, and age-related macular degeneration^5–7^.

Quantitative analysis of the choroid is hindered by shadows cast by anterior structures such as retinal vessels. In 2009, Fabritius *et al*. described a semi-automated method for estimating the shadows in the outer retina and choroid, and for adjusting the brightness of deep voxels to compensate for those shadows^8^. This was based on prior work by Duck and Hughes in the context of ultrasound^9^. In 2011, Girard *et al*. published a fully automated algorithm for OCT shadow compensation, which calculated a unique compensation factor for the intensity of each pixel, based on idiosyncrasies of OCT signal acquisition^10^. In 2013, the same group applied this technique to demonstrate enhanced visualization of the lamina cribrosa by OCT imaging^11^.

Although shadow compensation does not currently feature in the workflows of most viewing platforms which accompany commercially available OCT devices we believe that shadow compensation is important for successful and robust quantitative OCT analysis, especially of choroid and other sub-retinal pigment epithelial structures.

The purpose of this paper is to perform quantitative analysis of the choroid in normal subjects, using the CVI as an example parameter, with and without shadow compensation.

## Results

Twenty-seven eyes of 27 subjects were examined. Mean age was 31.33 years (SD 6.84) and 18 subjects were male. Mean subfoveal choroidal thickness was 358 (SD: 64.8) μm and mean choroid volume was 4.10 (SD: 0.87) mm^3^. The main set of outcome measures determined whether the final calculated choroidal vascularity index was influenced by shadow compensation or not. Images were also examined qualitatively to gain insights into the performance of the toolchain.

**Figure 1:**
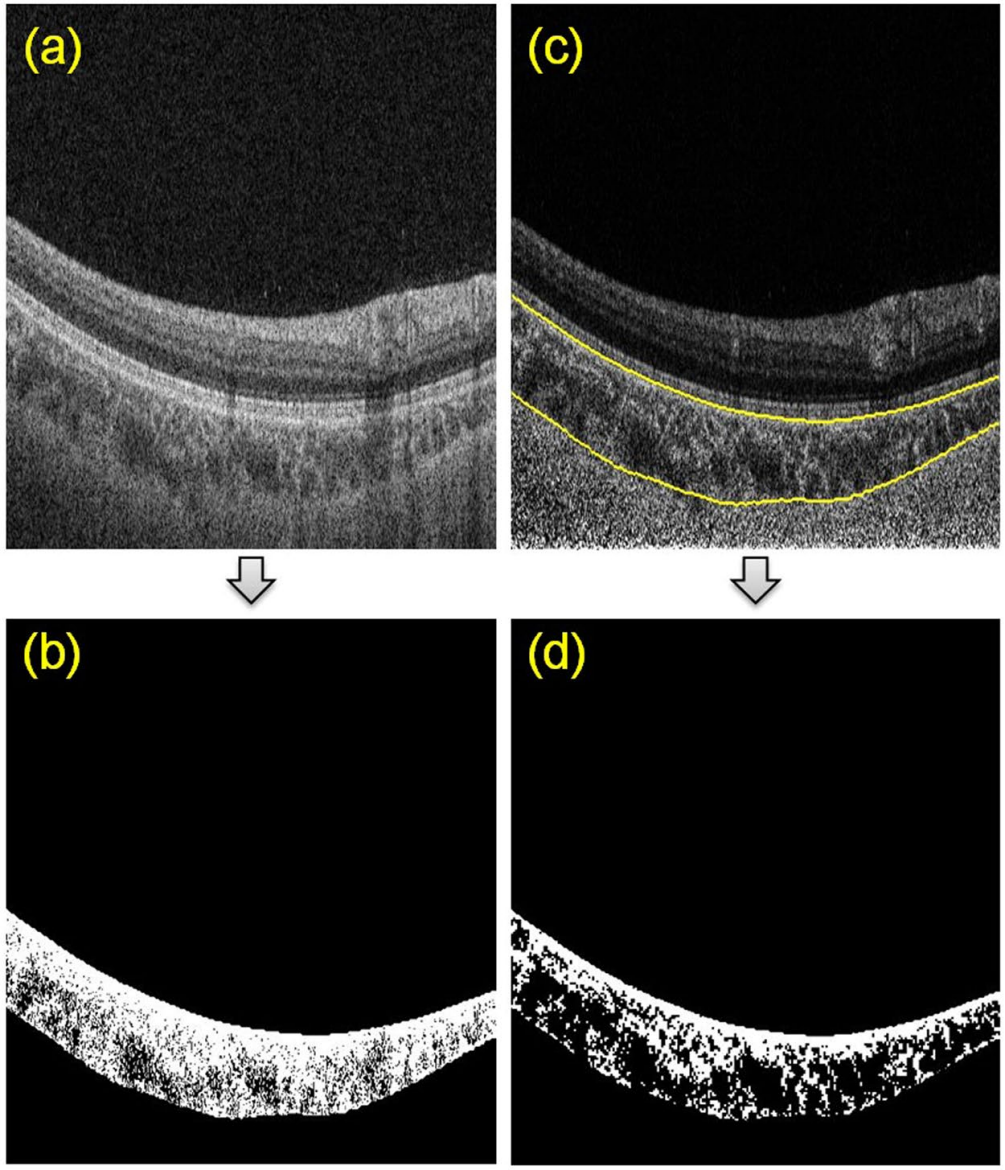
Comparison of choroid vascuature before and after shadow compensation and enhancement: (**a**) Raw OCT scan, (**b**) Binarized choroid layer applying traditional Otsu’s method on Raw OCT scan (**c**) Image Shadow compensated and enhanced image and (**d**) Binarized choroid layer using Vupparaboina *et al*.’s method obtained after shadow compensation an enhancement.

**Figure 2:**
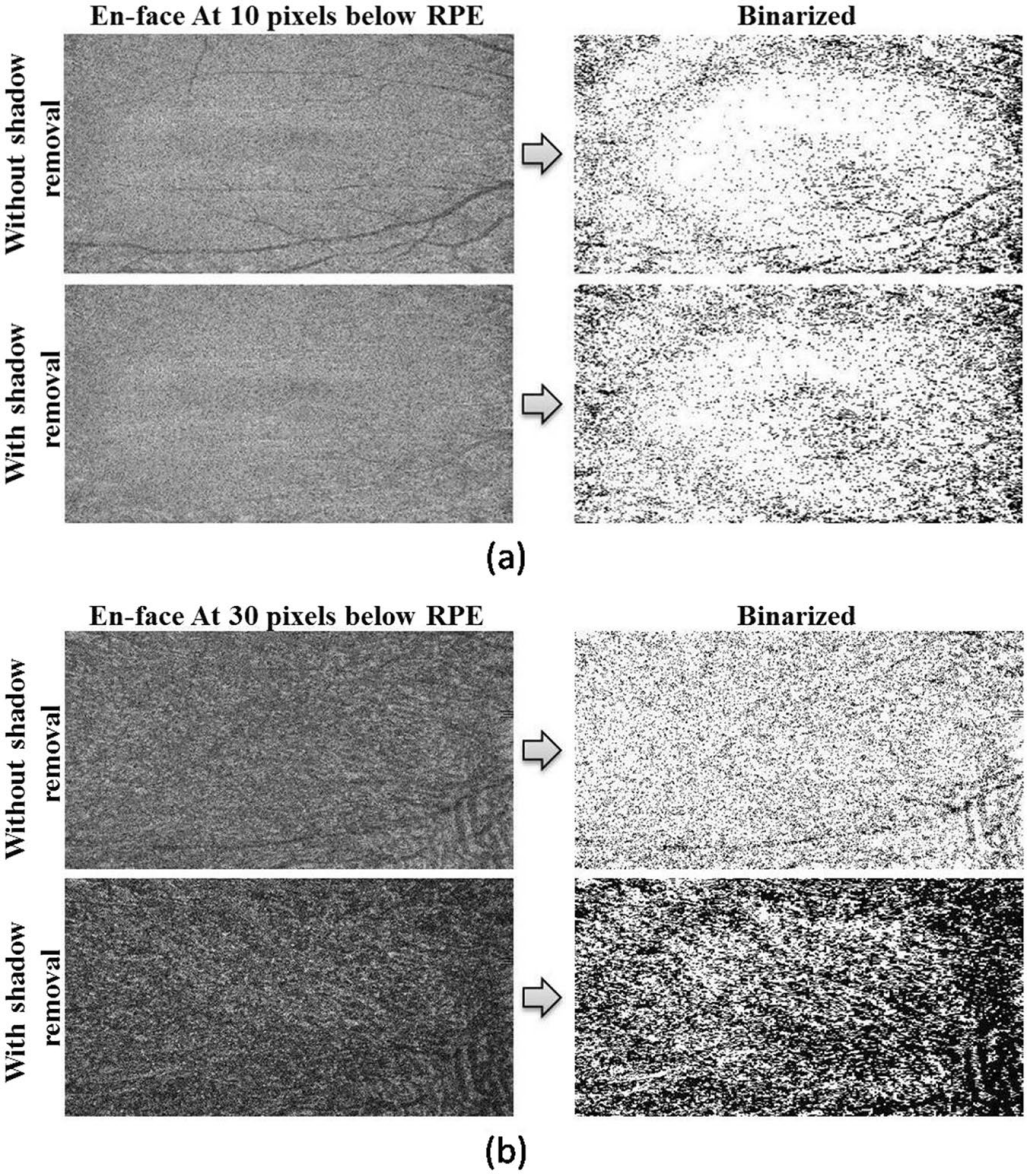
Before shadow compensation vs after shadow compensation, based on en-face images: (**a**) Effect of shadows of en-face images obtained at 10 pixels below RPEbefore (top) and after (bottom) shadow compensation with corresponding binarized images on the right; (**b**) Effect of shadows of en-face images obtained at 30 pixels below RPE before (top) and after (bottom) shadow compensation with corresponding binarized images on the right.

In shadow compensated OCT scans, the choroid was visualized with greater brightness and the masking of deep tissues by retinal blood vessels was greatly reduced. For a representative B-scan, Fig. 1 illustrates the improvement in choroid representation after shadow compensation. Figure 2 depicts a binarized *en face* OCT segments at two levels in the choroid before and after shadow compensation and enhancement. Qualitatively, luminal regions (dark regions) in binarized and shadow compensated choroid were consistent with corresponding regions in raw OCT scans; however those regions were not clearly detected in binarized choroid without shadow compensation.

In *en face* images taken at various depths below RPE, the effect of shadows of retinal vessels cast into the choroid were reduced after shadow compensation (Fig. 2). Furthermore, image enhancement improved the visualization of vasculature in binarized choroidal segments. Consequently, the calculated choroid vascularity index was significantly increased after shadow compensation and enhancement.

**Figure 3:**
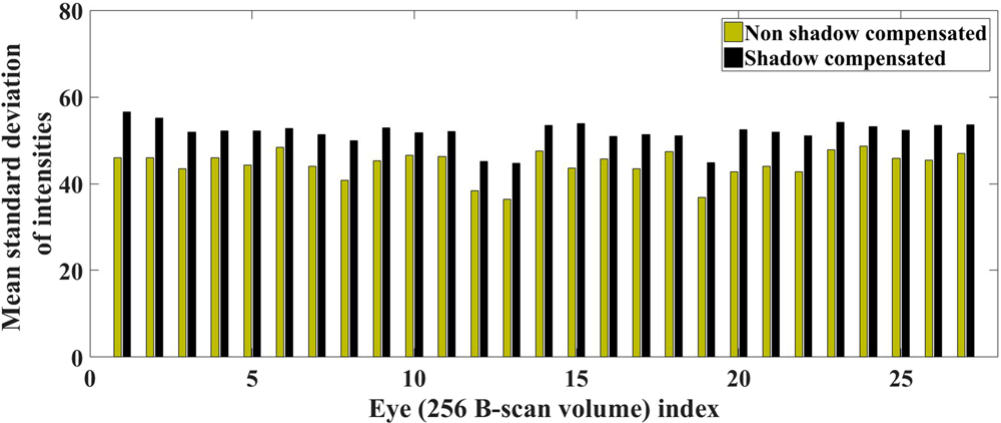
Comparison of contrast, interms of mean standard deviation of pixel intensities, measured for volume scans before and after shadow compensation.

The mean CVI measured for non-shadow compensated scans was 0.42 ± 0.12 (range 0.21–0.61), while the mean CVI measured for shadow compensated scans was 0.55 ± 0.02 (range 0.51–0.58). Coefficient of variation for CVIs was higher in non-shadow compensated scans (0.28), in comparison to shadow compensated scans (0.03), and this difference was statistically significant (p-value = 0.00; 95% CI −0.05).The decrease in variability after shadow compensation can be interpreted in two ways. One interpretation could be that there may be a low-pass filtering effect resulting by the image processing operations performed which smoothens the images. Consequently, this reduces the difference between the reflectivity profiles of stromal and luminal regions and hence decreases the variability in CVIs. Alternatively, the other interpretation could be that the contrast between stromal and luminal regions may be poor in raw images and may have increased significantly after performing shadow compensation and enhancement. Further, the increased contrast may be in agreement with actual tissue areas and thus the consistent CVIs. We claim that the later is the case in decreasing the variability, i.e., improved contrast between the stromal and luminal regions. To support the above claim, we measured the contrast of B-scan images before and after shadow compensation in terms of standard deviation of pixel intensities (see Fig. 3). For the 27 eyes at hand, we observed that standard deviation of pixel intensities for non shadow compensated scans varied between 36.37 and 48.68 with a mean 44.46 ± 3.23 while those for shadow compensated scans varied between 44.70 and 56.50 with a mean 51.71 ± 2.81, demonstrating the increase in contrast. Visual interpretation also indicated the same which we corroborate quantitatively based on observer grading, performed twice by two graders, as the baseline.

### Validation of observer grading

On the 27 eyes at hand, correlation coefficient (CC) indicating intra-observer repeatability, for the overall grading performed on B-scan images before and after shadow compensation was observed to be very high with values 99.46% and 99.91% for grader-A, and 97.98% and 98.56% for grader-B, respectively. Further, CC indicating obtained between grader-A and grader-B, indicating inter-observer repeatability, was observed to be 98.21% and 99.73%, respectively, before and after shadow compensation, demonstrating robust observer grading. More details on subjective grading including scores on selected B-scan images per subject are furnished in Table 1. Note that there appears a bias in intra observer scoring, which however do not impact the findings as the bias is consistent across images of both before and after shadow compensation.

**Table 1:** Subjective grading on binarized images obtained before and after shadow compensation. Notation: WOSR – Without shadow compensation; WSR – With shadow compensation; SD – Standard deviation.

	Grader	Data	Attempt	Mean (µ) (%)	SD (σ) (%)	Min (%)	Max (%)
Image-1	A	WOSR	1	57.59	10.59	30	70
			2	59.44	10.22	40	75
		WSR	1	85.37	5.53	70	95
			2	83.89	3.20	75	90
	B	WOSR	1	46.66	17.54	20	80
			2	54.07	16.41	30	75
		WSR	1	75.93	14.87	30	95
			2	82.96	9.22	60	90
Image-2	A	WOSR	1	49.81	10.60	30	65
			2	54.44	10.68	30	75
		WSR	1	84.81	5.27	70	95
			2	83.70	4.29	75	90
	B	WOSR	1	41.11	17.83	20	70
			2	45.00	18.86	20	70
		WSR	1	72.22	17.88	30	95
			2	80.92	9.91	60	90
Image-3	A	WOSR	1	47.22	10.59	30	65
			2	51.29	11.23	30	75
		WSR	1	83.70	6.14	70	95
			2	83.33	4.80	70	90
	B	WOSR	1	38.33	17.15	20	70
			2	43.33	17.15	20	75
		WSR	1	70.74	19.05	20	95
			2	81.29	8.50	60	90
Image-4	A	WOSR	1	52.96	9.83	35	70
			2	54.07	9.51	35	70
		WSR	1	85.18	4.04	75	95
			2	84.44	4.45	75	90
	B	WOSR	1	42.22	14.82	15	80
			2	46.29	14.97	30	80
		WSR	1	73.14	17.65	40	95
			2	83.89	5.43	70	90
Image-5	A	WOSR	1	53.89	10.12	35	70
			2	53.37	9.39	40	65
		WSR	1	85.00	5.88	70	95
			2	83.89	5.43	65	90
	B	WOSR	1	46.29	17.51	10	80
			2	50.00	13.87	20	80
		WSR	1	75.93	19.56	0	90
			2	83.33	15.50	10	90
Overall	**A**	**WOSR**	**1**	**55.37**	**9.19**	**35**	**70**
			**2**	**57.22**	**9.54**	**40**	**70**
		**WSR**	**1**	**85.74**	**3.85**	**80**	**95**
			**2**	**84.07**	**4.39**	**75**	**90**
	**B**	**WOSR**	**1**	**44.81**	**13.83**	**20**	**75**
			**2**	**48.88**	**13.68**	**30**	**75**
		**WSR**	**1**	**75.00**	**10.93**	**45**	**95**
			**2**	**83.33**	**6.93**	**65**	**95**

Similar observations are made for subjective grading performed on en face images. In particular, CC indicating intra observer repeatability, for the overall grading performed on en face images was observed to be 99.52% and 99.88% for grader-A, and 99.66% and 99.86% for grader-B, respectively. Further, CC indicating obtained between grader-A and grader-B, indicating inter-observer repeatability, was observed to be 99.52% and 99.88%, respectively.

### Quantitative analysis based on observer grading

**Figure 4:**
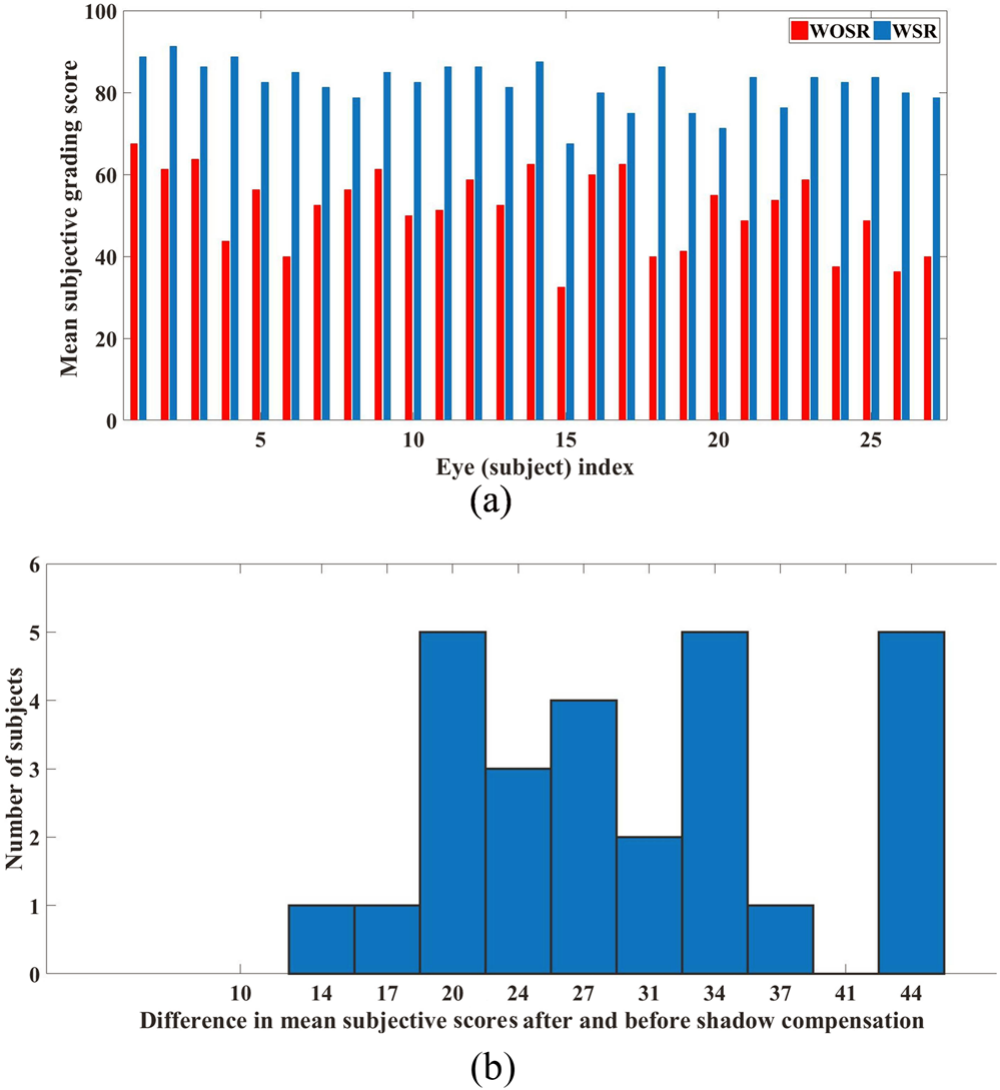
Subjective grading – before shadow compensation vs after shadow compensation: (**a**) Average subjective grading scores obtained twice by two masked graders before and after shadow compensation, and (**b**) Histogram of difference in mean subjective scores after and before shadow compensation. Notation: WOSR – Without shadow compensation; WSR – With shadow compensation.

In particular, based on the binarized images before shadow compensation, average grading (vascularity) scores obtained by all four attempts based on B-scans (twice by each grader per subject), observed to achieve a dynamic range between 32.50% and 67.50% with a mean 51.57% ± 9.70%, while the corresponding scores obtained after shadow compensation have a dynamic range between 67.50% and 91.25% with a mean 82.04% ± 5.53%, indicating significant improvement in identifying luminal regions (see Fig. 4a). Further, the difference in mean subjective grading before and after shadow compensation indicates that after shadow compensation and enhancement there was always an increase in the CVI (see Fig. 4b). Specifically, there was at least an increase of 12% with an average of 30%.

Analogously, based on en face images before shadow compensation, average grading values ranged between 51.20% and 72.50% with a mean 61.05% ± 4.97%, while the corresponding scores obtained after shadow compensation ranged between 77.50% and 95% with a mean 87.54% ± 3.64%.

### Comparing the CVIs estimated before and after shadow compensation

**Figure 5:**
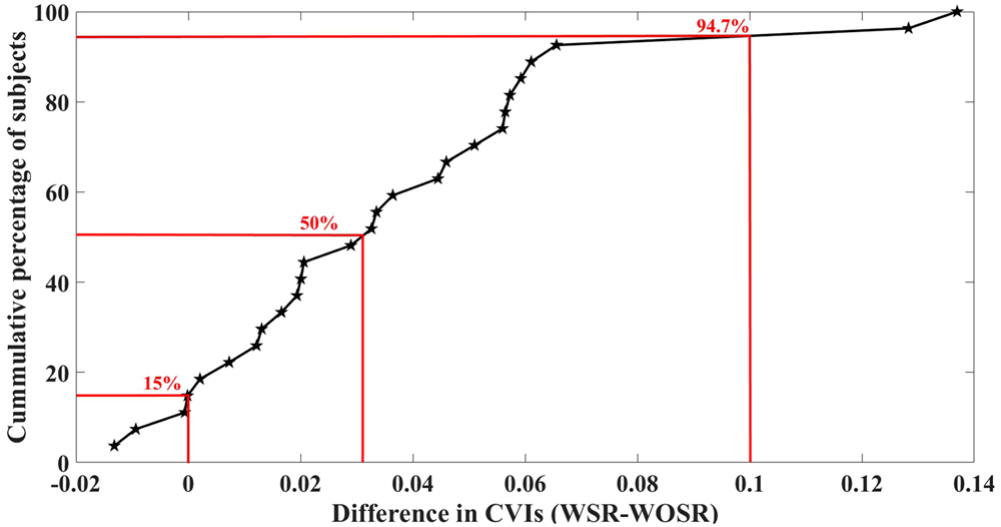
Comparison of difference in CVIs obtained for 27 eyes at hand before and after shadow compensation and enhancement.

**Figure 6:**
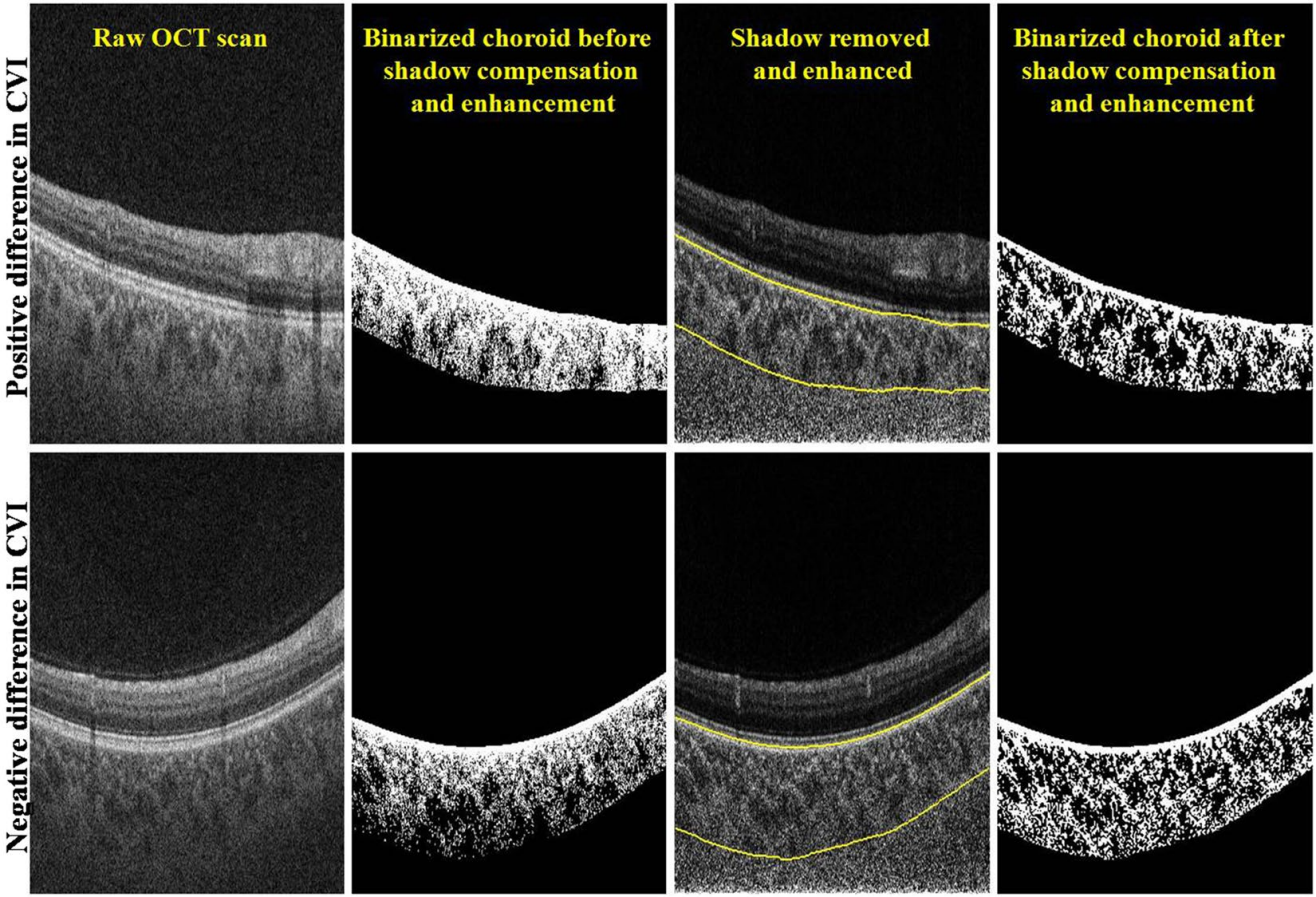
Comparison of vasculature based on difference in CVIs obtained before and after shadow compensation: Top—Instance of positive difference in CVI; and Bottom – Instance of negative difference in CVI.

For the 27 volume scans, Fig. 5 depicts the cumulative distribution of subjects plotted against the difference in CVIs estimated before and after shadow compensation. Specifically, in 94.7% of the subjects the difference in CVIs was up to 0.1; in 50% of the subjects the difference was up to 0.03; and in 15% of the subjects we observed a negative CVI. However, upon careful inspection of those volume scans which has negative difference in CVI, we observed that the thresholding is poor in images before shadow compensation, especially in the outer choroidal region. In particular, there exist large dark regions towards choroidal-sclera interface which perhaps is amounting to greater CVI. To see this, Fig. 6 depicts representative B-scans from volume scans with minimum (negative) and maximum (higher positive) CVIs. In summary, we have observed a mean improvement (increase) of 40% in CVI after shadow compensation and enhancement. Further, a statistically significant CVI difference (p-value) of 0.00 (95% CI −0.05) in paired t-test was observed.

## Discussion

Most of the published literature on imaging the choroid with OCT is focussed on qualitative grading of morphological features and quantification of choroidal thickness but there is increasing interest in deriving and validating quantifiable parameters, other than thickness, which might be more disease-specific.

In this study, we used customized software toolchains assembled from previously described image processing algorithms to calculate the CVI in normal subjects. The CVI is of particular interest because it is recognised qualitatively that the lumina of choroidal vessels appear hyporeflective on conventional OCT, while vessel walls and stroma are more hyperreflective. Our main purpose was to determine the value of shadow compensation which might be expected intuitively to reduce the CVI by increasing the brightness of most voxels and by amplifying noise. However, we found that shadow compensation increased the estimate of luminal volume and therefore increased the estimate of choroidal vascularity. This finding is reassuring because it suggests that shadow compensation outputs a dataset with improved contrast between vascular lumen and choroidal stroma.

Interestingly, we noted that there was significant reduction in variability in CVIs measurement after shadow compensation. In addition, reflections from the optically heterogeneous choroid are markedly brighter than those from the relatively transparent neurosensory retina in shadow compensated images, as would be expected based on our understanding of functional histology. Subjective grading suggests that shadow compensated scans demarcates the luminal and stromal regions better than the non shadow scans. Shadow-compensated scans may represent “true to the tissues” and represent absolute tissue reflectivity more accurately than unprocessed OCT scans. However, this needs to be substantiated with histological evidence.

We also note that, change in CVI presented in this study, itself may not be the ideal indicator of improvement in stromal and luminal regions. For instance, in the case zero difference in CVI, the respective stromal and luminal regions in binarized B-scans obtained before and after shadow compensation may not necessarily match. However, the subjective grading suggests that in general the quality is improved with shadow compensation.

As alluded earlier, we noted a bias in the subjective grading across observers which remained consistent across shadow compensated and non shadow compensated scans. This is expected because it is difficult for an observer to quantify his opinion on a discrete scale and may vary between observers. In other words, each observer’s good and bad rating may slightly differ which results in a bias. However, this bias does not affect the overall study as it is consistent for before and after shadow compensated scans.

A theoretical physical limitation imposed by OCT shadows is a reduction in the dynamic range of deeper reflectivity values. The images available to us were encoded as 8-bit grey scale, where each pixel or voxel is represented as an integer between 0 and 255. Shadows impose an even smaller range on deeper tissues, which might confound certain types of analysis. Practically however, the incorporation of shadow compensation by device manufacturers further upstream in the toolchain would enable them to use raw reflectivity data which is generally acquired with much greater (floating point) precision.

Application of shadow compensation could extend to optical coherence tomography angiography (OCTA) which uses short-time-lapse structural OCT data to derive the locations of dynamic voxels through decorrelation techniques and to infer blood flow^12^. Due to the relative transparency of ocular structures, decorrelation effects arising in relatively anterior voxels are projected onto relatively posterior hyperreflective structures. The appearance of projection artifacts in en face OCT angiographic segments of deep tissue confounds quantitative analysis of the deep retinal capillary plexus, and, potentially of neovascular tissue, necessitating projection resolution in software^13^. Shadow compensation performed prior to decorrelation analysis may be helpful in reducing projection artifacts and improving quantitative and qualitative analysis of OCTA data.

The main limitation of this study is that at present its scope is limited to normal subjects. However, we believe that it illustrates convincingly that shadows are expected artifacts of any depth-resolved imaging modality and that their effects should be understood by all clinicians and researchers embarking on quantitative analysis of OCT voxel data. Since the quantitative study of choroidal OCT is still in its relative infancy, we recommend that future studies in patients with pathology should include a shadow compensated study arm until its full implications are better characterized. As a step forward, we envision to test the performance of shadow compensation on diseased eyes specifically with irregular RPE and geographic atrophy.

## Methods

This observational study was conducted with the approval of the Hyderabad Eye Resaerch Foundation Ethics committee (institutional review board) and followed the tenets of the Declaration of Helsinki. Normal subjects between 19 and 47 years of age were recruited at the L.V. Prasad Eye Institute, Hyderabad India. All subjects provided informed consent for OCT imaging of the ocular posterior segment and analysis. Scan data were anonymized for analysis, as required by the Health Insurance Portability and Accountability Act of 1996.

Optical coherence tomography scans were acquired using a Triton swept-source OCT device (Topcon Corporation, Tokyo, Japan), which uses a tunable laser centered at 1050 nm to acquire 100,000 A scans per second. All scans were performed over a 6 × 6 mm^2^ area consisting of 256 un-averaged horizontal cross-sectional scans per acquisition. The resulting 3-dimensional voxel arrays were exported from Topcon’s viewing software as 8-bit grey scale image stacks.

**Figure 7:**
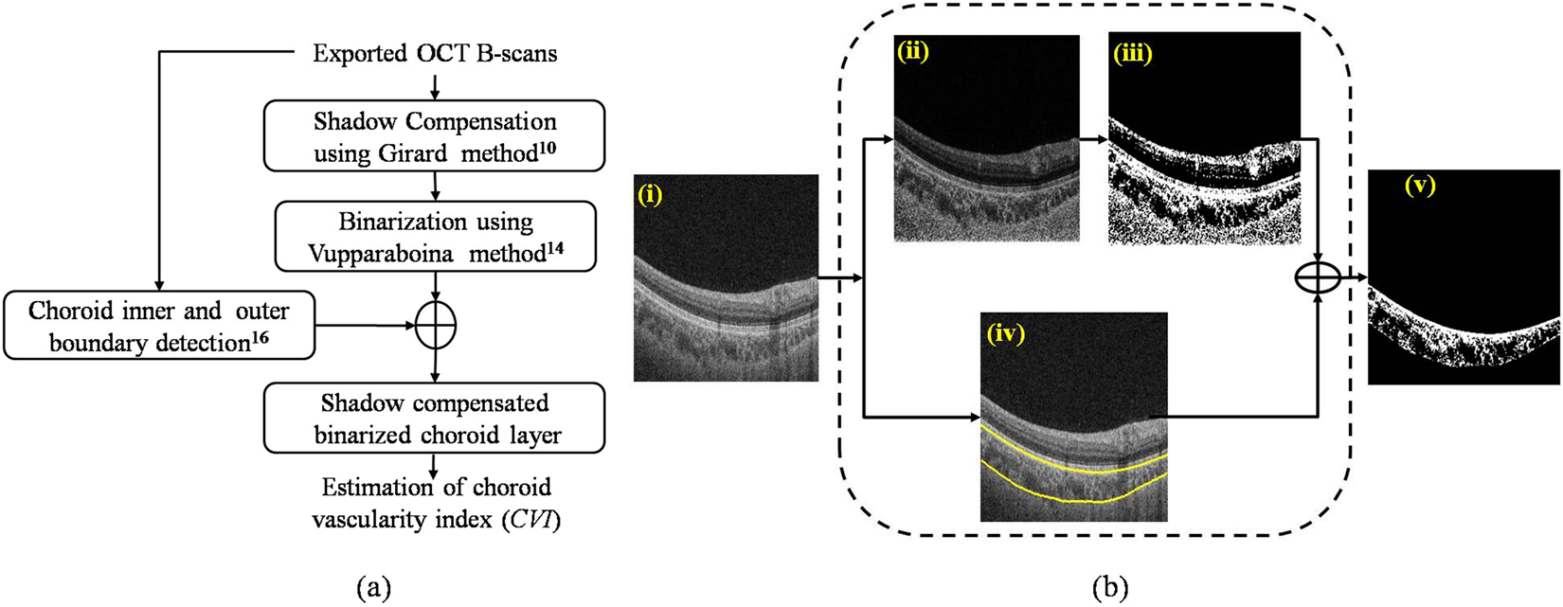
(**a**) Schematic of the proposed methodology; (**b**) Graphical representation of the proposed methodology: (i) Raw OCT B-scan, (ii) Shadow compensated and enhanced image, (iii) Image after binarization using Vupparaboina method^14^; (iv) Detected choroid boundaries and (v) Binarized choroid layer.

We developed a fully automated toolchains to obtain CVIs with and without shadow compensation. Our algorithm is depicted in Fig. 7a and each step is described below.

### Shadow Compensation

Shadow compensation was performed using the Girard algorithm on each of the 8-bit grey scale A-scans which comprise the volume data. Each A-scan is transformed into its raw intensity domain and each pixel intensity is multiplied by a unique compensation factor derived from idiosyncrasies of OCT signal acquisition. This method also enhances the contrast with which blood vessels are rendered. Figure 7b(ii) depicts the shadow compensated and enhanced image for the representative B-scan image shown in Fig. 7b(i).

### Binarization

Binarization of the OCT image facilitates quantification of choroidal luminal and stromal regions. We used a binarization method previously published by our group^14^. The first step is adaptive histogram equalization at the level of individual B-scans, considering 8 × 8 blocks, to increase contrast for choroid vessels. This is followed by exponential enhancement (exponentiation factor = 4, empirically obtained) on OCT B-scan images to increase the dynamic range of pixel intensities. A further non-linear enhancement is performed where each row of the resulting image is multiplied by the square of its row number, ensuring uniform distribution of pixel intensities among luminal and stromal regions. Finally, thresholding is performed using Otsu’s method^15^. An example binarized image is presented as Fig. 7b(iii).

### Choroid inner and outer boundary detection

At this point, we turn to define the inner and outer choroidal boundaries in the binarized image. We adopted the previously validated method published by our group, which performed with a Dice coefficient exceeding 95% in a direct comparison with manual segmentation. The Dice coefficient quantifies the correspondence of choroidal boundaries delineated by different strategies. The choroidal inner boundary was detected based on an intensity gradient at Bruch’s membrane and choroid outer boundary was detected using structural similarity index and tensor voting strategy^16^. The boundaries obtained for the representative image are depicted in Fig. 7b(iv). Finally, the detected choroid boundaries were used to segment out the choroid from the previously obtained binarized image (see Fig. 7b(v)). Notice that in the binarized choroidal layer, dark regions indicate the choroid vessel lumina and the white regions indicate the stroma.

### Choroid vascularity index (CVI)

The CVI was calculated from the binarized choroidal segments as the ratio:

$${\rm{CVI}}=\frac{\text{Volume}\,\text{of}\,\text{the}\,\text{choroidal}\,\text{luminal}\,\text{region}}{\text{Total}\,\text{volume}\,\text{of}\,\text{the}\,\text{choroid}}$$

### Observer grading for accuracy analysis

For evaluating the accuracy of the algorithm it is imperative to have ground truth segmentation such as manual labelling. However, in this case, it is challenging to have manual labelling due to the structural intricacies involved with the choroidal vascular cross-sections. Against this backdrop, we evaluated the accuracy of our proposed methodology based on observer grading to compare the stromal and luminal regions in choroid, obtained before and after shadow compensation. We recruited two graders (AG and MAR) to grade the segmented luminal and stormal region (grades 0–100%) in binarized choroid layer obtained before and after shadow compensation in masked fashion. To this end, two masked observers graded twice, on separate sessions, 5 B-scan images per eye (indexed–1, 64, 128, 192, 256 from 256 images) as well as 5 en face images per eye (taken at 5, 10 20 30 40 50 pixels below RPE) taken post binarization before and after shadow compensation. Three images, original and binarized B-scans are created as a montage placing side-by-side for grading. Both the observers were masked to their own as well as each other’s measurements. In addition to grading on individual scans from each eye, overall grading of the eye was also performed. For this, a montage of 15 images was created placing 5 images each of original and binarized scans before and after shadow compensation in three rows, top middle and bottom, respectively for B-scans. Similarly, a montage of 18 images was created for cumulative grading of en-face images. Two observations by each observer established the repeatability and the grading by two observers established the reproducibility of the grading method, respectively. To this end, we performed correlation coefficient (for two measurements *x*_*i*_ and *y*_*i*_, *i* = 1 …*N*, the correlation coefficient (${\rm{CC}})={\sum }_{i=1}^{N}{x}_{i}{y}_{i}/\sqrt{{\sum }_{i=1}^{N}{x}_{i}^{2}{\sum }_{i=1}^{N}{y}_{i}^{2}}$) analysis to evaluate repeatability of both intra- and inter-observer grading.

Finally, we considered the average of all the four observers’ grading to facilitate comprehensive evaluation between two methods.

### Statistical Analysis

CVIs obtained before and after shadow compensation were compared statistically. In particular, cumulative difference of CVIs obtained before and after shadow compensation was plotted. Subsequently, statistically significant difference (*p* value) at 95% confidence interval was also obtained. Further, variability in CVIs, obtained before and after shadow compensation, was also measured using the coefficient of variation i.e., ratio of standard deviation to mean.

### Implementation details

The individual operations were implemented as custom code in MATLAB (version 8.5.0.197613 (R2015a), MathWorks, Natick, MA) on a personal computer with Intel^®^ core i7 processor, 16 GB system memory, and Windows^®^ 7 Enterprise 64-bit operating system.

### Data availability

All data were available upon request. In case of any further information, Dr Jay Chhablani can be contacted at jay.chhablani@gmail.com.

## References

[1] Imamura Y, Fujiwara T, Margolis R, Spaide RF (2009). Enhanced depth imaging optical coherence tomography of the choroid in central serous chorioretinopathy. Retina.

[2] Copete, S., Flores-Moreno, I., Montero, J. A., Duker, J. S. & Ruiz-Moreno, J. M. Direct comparison of spectral-domain and swept-source OCT in the measurement of choroidal thickness in normal eyes. *British Journal of Ophthalmology*, bjophthalmol-2013-303904 (2013). 10.1136/bjophthalmol-2013-303904 24288394

[3] Gora M (2009). Ultra high-speed swept source OCT imaging of the anterior segment of human eye at 200 kHz with adjustable imaging range. Optics Express.

[4] Kuroda Y (2016). Increased choroidal vascularity in central serous chorioretinopathy quantified using swept-source optical coherence tomography. American journal of ophthalmology.

[5] Agrawal, R. *et al*. Influence of scanning area on choroidal vascularity index measurement using optical coherence tomography. *Acta Ophthalmologica* (2017). 10.1111/aos.13442 28470942

[6] Alshareef RA (2017). Subfoveal Choroidal Vascularity in Myopia: Evidence From Spectral-Domain Optical Coherence Tomography. Ophthalmic Surgery, Lasers and Imaging Retina.

[7] Ruiz-Medrano, J. *et al*. Age-related Changes In Choroidal Vascular Density Of Healthy Subjects Based On Image Binarization Of Swept-source Optical Coherence Tomography. *Retina* (2017). 10.1097/IAE.0000000000001571 28234809

[8] Fabritius T, Makita S, Hong Y, Myllylä R, Yasuno Y (2009). Automated retinal shadow compensation of optical coherence tomography images. Journal of biomedical optics.

[9] Hughes DI, Duck FA (1997). Automatic attenuation compensation for ultrasonic imaging. Ultrasound in medicine & biology.

[10] Girard MJ, Strouthidis NG, Ethier CR, Mari JM (2011). Shadow removal and contrast enhancement in optical coherence tomography images of the human optic nerve head. Investigative ophthalmology & visual science.

[11] Mari JM, Strouthidis NG, Park SC, Girard MJ (2013). Enhancement of Lamina Cribrosa Visibility in Optical Coherence Tomography Images Using Adaptive CompensationImproving Lamina Cribrosa Visibility in OCT Images. Investigative ophthalmology & visual science.

[12] Jia Y (2012). Split-spectrum amplitude-decorrelation angiography with optical coherence tomography. Optics express.

[13] Zhang M (2016). Projection-resolved optical coherence tomographic angiography. Biomedical optics express.

[14] Vupparaboina, K. K., Richhariya, A., Chhablani, J. & Jana, S. Optical coherence tomography imaging: Automated binarization of choroid for stromal-luminal analysis. *IEEE International Conference on Signal and Information Processing (IConSIP)*, 1–5 (2016).

[15] Otsu N (1979). A threshold selection method from gray-level histograms. IEEE transactions on systems, man, and cybernetics.

[16] Vupparaboina KK, Nizampatnam S, Chhablani J, Richhariya A, Jana S (2015). Automated estimation of choroidal thickness distribution and volume based on OCT images of posterior visual section. Computerized Medical Imaging and Graphics.

