# Author Correction: Quantitative shadow compensated optical coherence tomography of choroidal vasculature

**DOI:** 10.1038/s41598-018-27006-y

**Published:** 2018-06-07

**Authors:** Kiran Kumar Vupparaboina, Kunal K. Dansingani, Abhilash Goud, Mohammed Abdul Rasheed, Fayez Jawed, Soumya Jana, Ashutosh Richhariya, K. Bailey Freund, Jay Chhablani

**Affiliations:** 1 Surjana Center for Innovation, LV Prasad Eye Institute Hyderabad, Hyderabad, Telangana India; 2 Department of Electrical Engineering, Indian Institute of Technology Hyderabad, Hyderabad, Telangana India; 3 Department of Ophthalmology, University of Pittsburgh Medical Center, Pittsburgh, Pennsylvania USA; 4 Clinical Research, LV Prasad Eye Institute Hyderabad, Hyderabad, Telangana India; 5 Vitreo-retinal Service, LV Prasad Eye Institute Hyderabad, Hyderabad, Telangana India; 6 Truhlsen Eye Institute, University of Nebraska Medical Center, Omaha, NE United States; 7 Vitreous Retina Macula Consultants of New York, New York, New York, USA; 8 LuEsther T. Mertz Retinal Research Center, Manhattan Eye, Ear and Throat Hospital, New York, New York, USA

Correction to: *Scientific Reports* 10.1038/s41598-018-24577-8, published online 24 April 2018

The original version of this Article contained a typographical error in the name of the author K. Bailey Freund, which was incorrectly given as Bailey K. Freund. This has now been corrected in the PDF and HTML versions of the Article.

The Author Contributions section now reads:

“Conception and design: K.K.V.; K.K.D.; S.J.; A.R.; K.B.F.; J.C. Data collection: A.G. Analysis and interpretation: K.K.V.; K.K.D.; A.G.; M.A.R.; M.F.J.; S.J.; A.R.; K.B.F.; J.C. Writing the article: K.K.V.; K.K.D.; A.G.; S.J.; J.C. Critical revision of the article: K.K.D.; S.J.; A.R.; K.B.F.; J.C. Final approval of the article: K.K.V.; K.K.D.; A.G.; M.A.R.; M.F.J.; S.J.; A.R.; K.B.F.; J.C.”

This has now been corrected in the PDF and HTML versions of the Article.

